# Effects of Virtual Reality-Based Physical and Cognitive Training on Executive Function and Dual-Task Gait Performance in Older Adults With Mild Cognitive Impairment: A Randomized Control Trial

**DOI:** 10.3389/fnagi.2019.00162

**Published:** 2019-07-16

**Authors:** Ying-Yi Liao, I-Hsuan Chen, Yi-Jia Lin, Yue Chen, Wei-Chun Hsu

**Affiliations:** ^1^Department of Gerontological Health Care, National Taipei University of Nursing and Health Sciences, Taipei, Taiwan; ^2^Department of Physical Therapy, Fooyin University, Kaohsiung, Taiwan; ^3^Graduate Institute of Biomedical Engineering, National Taiwan University of Science and Technology, Taipei, Taiwan

**Keywords:** dual-task gait, MCI, virtual reality, executive function, combined physical and cognitive training

## Abstract

**Background**: Walking while performing cognitive and motor tasks simultaneously interferes with gait performance and may lead to falls in older adults with mild cognitive impairment (MCI). Executive function, which seems to play a key role in dual-task gait performance, can be improved by combined physical and cognitive training. Virtual reality (VR) has the potential to assist rehabilitation, and its effect on physical and cognitive function requires further investigation. The purpose of this study was to assess the effects of VR-based physical and cognitive training on executive function and dual-task gait performance in older adults with MCI, as well as to compare VR-based physical and cognitive training with traditional combined physical and cognitive training.

**Method**: Thirty-four community-dwelling older adults with MCI were randomly assigned into either a VR-based physical and cognitive training (VR) group or a combined traditional physical and cognitive training (CPC) group for 36 sessions over 12 weeks. Outcome measures included executive function [Stroop Color and Word Test (SCWT) and trail making test (TMT) A and B], gait performance (gait speed, stride length, and cadence) and dual-task costs (DTCs). Walking tasks were performed during single-task walking, walking while performing serial subtraction (cognitive dual task), and walking while carrying a tray (motor dual task). The GAIT Up system was used to evaluate gait parameters including speed, stride length, cadence and DTCs. DTC were defined as 100 * (single-task gait parameters − dual-task gait parameters)/single-task gait parameters.

**Results**: Both groups showed significant improvements in the SCWT and single-task and motor dual-task gait performance measures. However, only the VR group showed improvements in cognitive dual-task gait performance and the DTC of cadence. Moreover, the VR group showed more improvements than the CPC group in the TMT-B and DTC of cadence with borderline significances.

**Conclusion**: A 12-week VR-based physical and cognitive training program led to significant improvements in dual-task gait performance in older adults with MCI, which may be attributed to improvements in executive function.

## Introduction

Performing activities of daily living (ADLs) requires the ability to perform multiple cognitive and physical tasks simultaneously. Dual tasking destabilizes gait performance, especially for those with impaired cognitive function, which may lead to falls (Springer et al., 2006). A previous study reported that older adults with mild cognitive impairment (MCI) showed significant decreases in gait velocity, increases in stride time and increases in stride time variability when changing from a single to a dual task (Muir et al., 2012). Meta-analysis has also revealed some differences between MCI patients and cognitively normal controls in several gait parameters, including velocity, stride length and stride time, during either a single or dual task (Bahureksa et al., 2017). In addition, dual-task gait performance has been associated with progression to dementia in patients with MCI. Dual-task gait testing may be used by clinicians to assess the risk of cognitive decline (Montero-Odasso et al., 2017; Rosso et al., 2019).

Executive function is defined as a set of cognitive skills necessary for planning, monitoring and executing a sequence of goal-directed complex actions (Diamond, 2013). Executive dysfunction, such as inadequate divided attention and selective attention, are more pronounced in older adults with MCI than in controls (Johns et al., 2012; Kirova et al., 2015). Executive function can modulate competition interference between two attention-demanding tasks and has been suggested to be associated with spatial and temporal characteristics of dual-task gait performance (Woollacott and Shumway-Cook, 2002; de Bruin and Schmidt, 2010). For example, Doi et al.’s (2014) study showed significant correlation between executive function and dual task gait speed in 389 older adults with MCI. Consequently, individuals with poor executive function have reduced gait speed and higher levels of fall risk and functional disability (Johnson et al., 2007; Herman et al., 2010). Improving executive function and minimizing dual-task interference may, therefore, have clinical utility in avoiding falls occurring in older adults with MCI (Bahureksa et al., 2017).

Either cognitive or physical training has proven to be an effective intervention in enhancing cognitive functions in older adults with MCI (Simon et al., 2012; Suzuki et al., 2012, 2013). Therefore, some studies have investigated the combined effects of physical and cognitive training (Barnes et al., 2013; Anderson-Hanley et al., 2018; Damirchi et al., 2018). Studies have found that older adults with MCI showed greater improvements in various cognitive functions after receiving combined therapy than after receiving cognitive or physical therapy alone (Barnes et al., 2013). In addition to improved cognitive function, dual-task gait interference may be decreased through the repeated practice of dual tasking (i.e., combined physical and cognitive interventions) in accordance with the principles of task-specific training (Plummer et al., 2015). However, the amount of evidence on the effects of combined physical and cognitive exercises on dual-task gait performance in older adults with MCI is limited. Tay et al.’s (2016) finding showed that combined physical and cognitive training improved dual-task walking performance. More evidence is required to investigate the training effects in older adults with MCI.

Virtual reality (VR) is a computer-generated technology that enables interactions between the user and virtual environments. The advantages of using VR interventions include enhancing accessibility and cost-effectiveness, creating an immersive experience, and providing immediate feedback based on an individual’s performance. Previous articles have shown positive effects of these VR interventions on attention and visual and verbal memory, as well as executive functioning, in older adults with MCI (Coyle et al., 2015; Mrakic-Sposta et al., 2018). Due to the advantages of VR, integrating physical and cognitive training into VR appears to be a good intervention approach. However, most articles using VR training include either physical or cognitive training (García-Betances et al., 2015). Studies on the effects of combining both physical and cognitive training in the VR context are lacking. In addition, instrumental ADLs (IADLs) comprise many dual-task activities and require high demands on executive functioning. Integrating IADL tasks into the VR context represents an innovative approach to improve executive function in older adults with MCI. Whether these innovative interventions can reduce dual-task interference and transfer to improvements in dual-task gait performance requires investigation. Therefore, the purpose of the current study was to assess the effects of VR-based physical and cognitive training on executive function and dual-task gait performance in older adults with MCI, as well as compare the VR-based physical and cognitive training with traditional combined physical and cognitive training.

## Methods

### Study Design and Protocol

This study was a single-blinded (assessor) randomized controlled trial. Participants were randomly assigned to either the VR training group or the combined physical and cognitive training (CPC) group *via* a sealed envelope. Subjects in the VR group participated in a 60-min, VR-based physical and cognitive training each visit, three times a week for 12 weeks. Those in the CPC group participated in combined physical and cognitive training for 60 min each visit, three times a week for 12 weeks. An experienced physical therapist supervised exercise training in a small group for both the VR and CPC groups. The assessor, who was always blinded to the group assignments, measured the outcomes at baseline and after completing the 36 sessions. This trial was approved by the Institutional Review Board of National Yang-Ming University, and consent forms were obtained from all participants at the beginning of the experimental procedures. The trial was registered in the Thai Clinical Trials Registry^1^, and the approval number is “TCTR20180531001.”

### Participants

All participants were recruited from communities and day care centers of Taipei, Taiwan. The inclusion criteria were: (1) aged 65 years and over; (2) able to walk more than 10 m without walking aids; (3) had a Montreal Cognitive Assessment (MoCA) score lower than 26 (Tsai et al., 2012); (4) had self-reported memory complaints; and (5) had the ability to perform ADLs. The exclusion criteria included: (1) dementia; (2) a history of malignant tumors with life expectancy less than 3 months; (3) the presence of an unstable neurological or orthopedic disease interfering with participation in the study; and (4) an education level less than 6 years (elementary school).

### Intervention

#### Combined Physical and Cognitive Training (CPC) Group

Our CPC program contains both physical and cognitive elements of training. The physical training regimen comprised resistance, aerobic and balance exercises that meet the standards of the American College of Sports Medicine for seniors (Chodzko-Zajko et al., 2009; Garber et al., 2011). Our physical training was set to reach 50%–75% of the maximal heart rate (calculated as 220- age) with the exertion perceived by the participants as “somewhat hard” (scored 13–14). Specifically, Therabands were applied to assist the training of both the upper and lower extremities during the resistance exercise. A series of whole-body aerobic exercises, for example, stepping while in the seated and standing positions, as well as on and off of a stool, was performed. Balance exercises included standing on a steady foam mat in various postures and walking forward and backward with eyes open and closed. Other functional tasks simulated ADLs and were designed to enhance motor performance and were integrated into the CPC program. Samples of functional tasks included asking participants to climb stairs, cross obstacles while reaching for objects, and turning and rising from a chair. In addition to the functional tasks, training that targeted cognitive abilities was also integrated into the physical training program. Training scenarios included walking while reciting poems, naming flowers and animals while crossing obstacles, solving math questions during the resistance training, drawing a circle in the air in the clockwise or counterclockwise direction with the right or left hand, respectively, and searching for the prefix and roots of a Chinese character at moments when they repetitively stand up from a chair.

#### Virtual Reality-Based Physical and Cognitive Training (VR) Group

**Figure 1:**
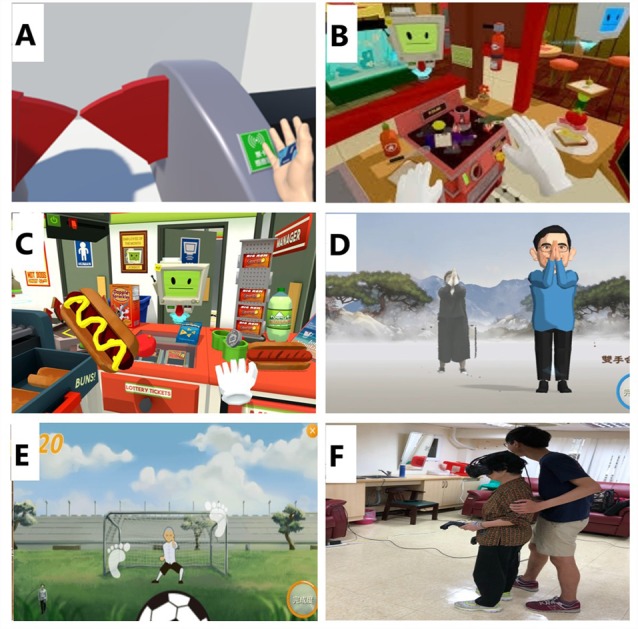
Scenes from the VR-based training program. **(A)** Take the MRT. **(B)** Kitchen chef. **(C)** Convenience store clerk. **(D)** Tai Chi. **(E)** Football (running and stepping). **(F)** Subject wearing VR glasses and performing VR tasks. **(B)** and **(C)** are derived from “job simulator” created by Owlchemy Labs. Both the subject and trainer have provided written informed consent allowing the publication of this image.

Scenes from the VR-based physical and cognitive training program are shown in Figure 1.

##### VR-Based Physical Exercise Program

We used the Kinect system (Microsoft Corporation, Redmond, WA, USA) to capture the limb motions and create a full-body 3D virtual map. The physical elements of the VR training were developed by the well-established and widely used Tano and LongGood programs. We adopted programs, including a simplified 24-form Yang-style Tai Chi, resistance exercise, aerobic exercise, and functional tasks in the forms of window cleaning, goldfish scooping and other tasks relevant to daily activities, to improve upper and lower extremity balance, stability, strength and endurance (Figures 1D,E). In the VR context, participants would imitate the virtual character and adjust their movements based on the simultaneous visual and auditory feedback.

##### VR-Based Cognitive Training Program

The cognitive training required wearing the VR glasses on their heads with a motor controller in both hands to execute the training tasks (Figure 1F). Our laboratory invented most of the cognitive training VR games, while others were derived from the “Job Simulator” software developed by Owlchemy Labs. The concept of the cognitive programs was inspired by simulated IADL tasks. For example, in the taking mass rapid transit (MRT) game, participants took the MRT in a familiar VR context where station gates, ticket vending machines, and ATMs were located in the usual places. To complete the task, a participant needed to be aware of their present location and the designated stations. They also needed to gather enough coins based on the fare chart to obtain a ticket. In the store finder game, a big red cross sign appeared as an indicator when something was going wrong. A participant needed to virtually walk to the store noted on a map in less than 3 min. If the participants failed to get closer to the targeted store in 2 min, directional marks in red popped up to guide their way. In the kitchen chef game, a participant found herself/himself in a well-equipped kitchen surrounded by numerous utensils available for use to prepare an ordered dish. Once he or she was able to complete a simple meal, a more complicated dish requiring more ingredients and utensils to complete followed. The last game was convenience store clerk; participants were responsible for gathering items from the to-do list and checking them out. Some of the listed items were easy to find, while others could not be located as easily (Figures 1A–C).

### Outcome Measures

These outcomes were all secondary outcomes of our project. We wanted to explore how our secondary outcomes are responded to the intervention. Therefore, this is an exploratory study of mechanisms.

### Executive Function

#### Trail Making Test (TMT)

The TMT has been hypothesized to reflect components of executive function, such as visual attention and task switching (Arnett and Labovitz, 1995). The current study used TMT-A and the Chinese version of TMT-B (Wang et al., 2018b). The TMT-A was composed of 25 consecutive Arabic numbers, and participants connected the numbers while following the numerical sequence. The TMT-B was composed of 12 consecutive Arabic numbers and 12 Chinese characters that represented a Chinese zodiac sign. Participants drew lines to connect the circles in ascending order while following an additional rule of alternating between numbers and animal signs (i.e., 1 - rat - 2 - ox - 3 - tiger, etc.). The time to complete each test was recorded. Delta TMT (TMT B subtract TMT A) was also recorded as our TMT outcome.

#### Stroop Color and Word Test (SCWT)

The Stroop Color and Word Test (SCWT) has been used to assess the ability of inhibition in executive function (Scarpina and Tagini, 2017). The current study used the Chinese version of the Stroop (Wang et al., 2018b), which was composed of four characters and four colors. The incongruous conditions were used in the present study. In this condition, the colors of the characters were printed in an inconsistent color ink (e.g., the character “blue” in red ink). Participants had to name the color of the ink rather than state the word/character as quickly as they could in a limited time. The number of correct answers in 45 s (SCWT number) and time to name 45 characters (SCWT time) were our outcomes.

### Gait Performance

Gait performance was measured in three conditions: (1) walking at their preferred walking speed (single task); (2) walking while executing a serial subtraction by three task, starting from a randomized 3-digit number (e.g., 100, 97, 94…; cognitive dual task); and (3) walking while carrying a tray with glasses of water (motor dual task). Our primary task is waking, and the secondary tasks are executing a serial subtraction or carrying a tray. Participants were asked to focus on their walking task and walk three trials under each condition. The trial intervals were 1 min. Data were averaged from the three trials.

The GAIT Up system (Gait Up, Lausanne, Switzerland), a wearable device with good validity and reliability, was used to evaluate gait parameters for the above testing conditions (Mariani et al., 2010; Dadashi et al., 2014). The dimensions of the GAIT UP sensors were 50 mm × 40 mm × 16 mm, and the weight was 36 g. Two wireless inertial sensors with tri-axial accelerometers were fixed on the upper part of the shoe with an elastic strap. In the present study, spatiotemporal parameters recorded during each trial included speed (m/s), stride length (cm) and cadence (step/min). Dual-task interference was quantified by calculating the dual-task costs (DTCs) according to the customary formula (McDowd, 1986). For example, DTC-speed [%] = 100 * (single-task walking speed − dual-task walking speed)/single-task walking speed.

### Data Analysis

Demographic and behavioral data analyses were performed using SPSS 20.0 software (SPSS Inc., Chicago, IL, USA). Descriptive statistics were generated for all variables, and the distributions of the variables were expressed as the means ± standard deviations or as the numbers (%). Intergroup differences in the baseline characteristics were analyzed using independent *t*-tests or chi-square tests. Two-way analysis of variance (ANOVA) with repeated measures was used to determine the effects of the intervention on executive function and gait performance. The model effects were the groups (VR and CPC), the times (pre and post), and their interaction. The intergroup comparison was across groups, and the intragroup comparison was the change over time. A *post hoc* Tukey’s test was used for variables with group × time interaction effects.

## Results

**Figure 2:**
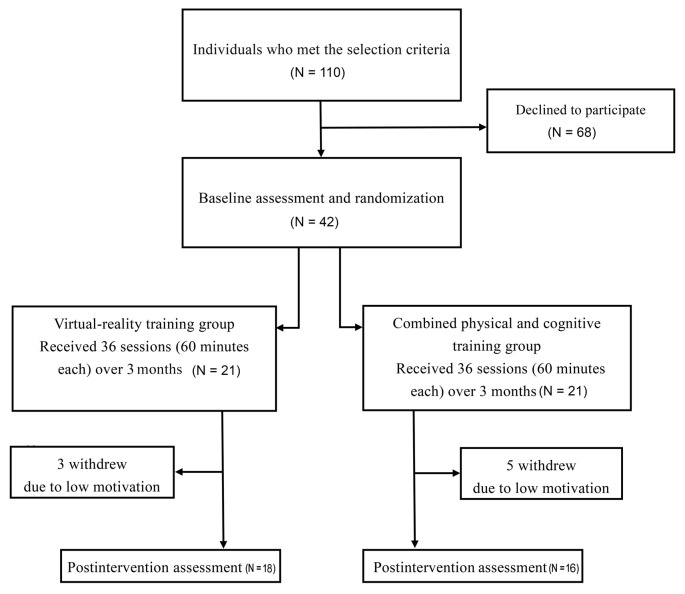
Flowchart of the present study.

As shown in the flowchart, 42 participants were recruited and randomly assigned to either the VR group (*n* = 21) or the CPC group (*n* = 21). Three participants in the VR group and five participants in the CPC group dropped out due to low motivation. A total of 34 participants (18 in the VR group and 16 in the CPC group) completed all the assessments (Figure 2). No adverse events were reported throughout the study period. Participant characteristics are shown in Tables s 1–3. Baseline demographic characteristics and outcome measures at the pre-intervention are similar between two groups.

**Table 1:** Baseline demographic characteristics of the participants (*N* = 34).

	Virtual reality training group (*N* = 18)	Combined physical and cognitive training group (*N* = 16)
Age (years)	75.5 ± 5.2	73.1 ± 6.8
Sex (female/male)	11/7	12/4
Height (cm)	159.0 ± 9.7	155.4 ± 7.1
Body weight (kg)	61.3 ± 8.5	56.0 ± 9.5
BMI (kg/m^2^)	24.4 ± 4.2	23.2 ± 3.6
Education (years)	9.3 ± 3.8	9.9 ± 2.1
MMSE (score)	27.2 ± 1.9 (range: 18–30)	27.2 ± 1.6 (range: 17–29)
MoCA (score)	22.84 ± 2.69 (range: 18–26)	23.15 ± 2.96 (range: 17–26)

*MMSE, Mini-Mental State Examination; MOCA, Montreal Cognitive Assessment. The data are presented as the means ± SDs or numbers*.

**Table 2:** Comparison of executive function in the virtual reality (VR) training group and the combined physical and cognitive training group.

	VR group (*n* = 18)			CPC group (*n* = 16)			Between-group difference, *p*	
	Pre-intervention	Post-intervention	Within-group difference, *p*	Pre-intervention	Post-intervention	Within-group difference, *p*	Baseline	Time × Group Interaction
**Trail Making Test (TMT)**								
TMT-A (s)	75.77 ± 34.95	66.00 ± 22.38	0.088	71.50 ± 27.30	64.31 ± 23.43	0.099	0.935	0.710
TMT-B (s)	179.22 ± 58.06	134.21 ± 48.23	<0.001*	154.50 ± 63.50	136.37 ± 48.58	0.098	0.792	0.032*
Delta TMT (s)	103.44 ± 51.60	68.61 ± 45.22	<0.001*	83.00 ± 45.72	72.06 ± 39.70	0.334	0.233	0.065
**Stroop Color and Word Test (SCWT)**								
SWCT-numbers *(n)*	15.05 ± 6.59	19.44 ± 9.05	0.003*	16.87 ± 7.72	20.21 ± 8.53	0.029*	0.932	0.835
SWCT- time (s)	126.83 ± 41.03	100.66 ± 33.93	<0.001*	119.87 ± 54.35	100.18 ± 41.89	0.002*	0.648	0.315

TMT-A, Trail Making Test, part A; TMT-B, Trail Making Test, part B; Delta TMT, TMT-B minus TMT-A; SCWT, Stroop Color and Word Test; SCWT-number, The number of correct answers in 45 s; SCWT-time, time to name 45 characters. The data are presented as the means ± SDs or numbers. *Significance level <0.05.

**Table 3:** Comparisons of single-task and dual-task gait performance in the VR training group and the combined physical and cognitive training group.

	VR group (*n* = 18)			CPC group (*n* = 16)			Between-group difference, *p*	
	Pre-intervention	Post-intervention	Within-group difference, *p*	Pre-intervention	Post-intervention	Within-group difference, *p*	Baseline	Time × Group Interaction

**Single-task gait**								
Speed (cm/s)	82.3 ± 29.1	92.9 ± 28.5	0.016*	89.3 ± 23.3	100.19 ± 25.7	0.047*	0.450	0.971
Stride length (cm/s)	89.5 ± 24.6	98.6 ± 27.4	0.018*	93.6 ± 23.2	100.6 ± 20.0	0.082	0.620	0.671
Cadence (step/min)	113.1 ± 18.3	110.3 ± 30.9	0.713	116.8 ± 10.8	126.4 ± 13.2	0.002*	0.486	0.141
**Cognitive dual-task gait**								
Speed (cm/s)	68.1 ± 26.9	82.5 ± 30.6	0.003*	72.8 ± 25.9	78.1 ± 33.2	0.247	0.609	0.159
Dual-task costs: speed (%)	15.8 ± 14.8	12.1 ± 12.0	0.231	19.07 ± 14.0	24.39 ± 28.2	0.469	0.517	0.235
Stride length (cm/s)	84.2 ± 25.6	96.2 ± 30.9	0.001*	80.7 ± 31.4	90.3 ± 22.5	0.221	0.718	0.761
Dual-task costs: stride length (%)	5.1 ± 12.5	3.2 ± 9.7	0.444	4.6 ± 19.3	10.5 ± 13.1	0.309	0.918	0.192
Cadence (step/min)	99.1 ± 16.4	103.1 ± 31.7	0.603	104.5 ± 17.3	109.9 ± 22.1	0.188	0.363	0.876
Dual-task costs: cadence (%)	0.11 ± 0.08	0.06 ± 0.07	0.024*	0.10 ± 0.10	0.13 ± 0.12	0.284	0.717	0.018*
**Motor dual-task gait**								
Speed (cm/s)	79.9 ± 29.9	92.3 ± 32.8	0.002*	86.5 ± 25.0	96.1 ± 27.3	0.008*	0.498	0.562
Dual-task costs: speed (%)	3.2 ± 8.1	1.8 ± 8.7	0.536	2.65 ± 17.0	4.13 ± 12.9	0.770	0.896	0.582
Stride length (cm/s)	86.5 ± 26.5	95.8 ± 29.6	0.003*	91.8 ± 20.7	96.7 ± 22.4	0.014*	0.520	0.183
Dual-task costs: stride length (%)	3.8 ± 8.8	3.5 ± 6.8	0.925	1.3 ± 13.6	4.2 ± 8.3	0.296	0.353	0.334
Cadence (step/min)	113.4 ± 16.0	112.1 ± 32.0	0.820	115.1 ± 15.1	125.0 ± 15.1	0.027*	0.799	0.170
Dual-task costs: cadence (%)	-0.01 ± 0.06	-0.01 ± 0.03	0.812	0.01 ± 0.06	0.01 ± 0.04	0.813	0.237	0.960

Dual task costs [%] = 100 * (single-task gait parameters − dual-task gait parameters)/single-task gait parameters. The data are presented as the means ± SDs. *Significance level <0.05.

The results of the executive function assessments are shown in Table 2. Of the six within group *p*-values for the TMT, VR group shows two significant values (TMT-B. delta TMT). None of the outcomes of TMT were found to have group × time interactions except for the TMT-B (a borderline significant *p* = 0.032). Of the four within group *p-values* for SCWT, both VR and CPC are significant (SWCT-numbers, SWCT-time); neither interaction is significant. The results of the single and dual task gait performance are shown in Table 3. For single-task gait, both groups have two significant *p-values* of 12 within group *p-values* (VR group: gait speed, stride length; CPC group: gait speed, cadence) and no interaction for between groups. For motor dual task gait, VR group has two significant within group *p-values* (gait speed, stride length), CPC group has three significant within group *p-values* (gait speed, stride length, cadence) of the 12 within group *p-values*, and none of the six interactions are significant. For cognitive dual task, VR group has three significant *p-values* of the 12 within group *p-values* (gait speed, stride length, DTCs of cadence), CPC group has no significant within group *p*-value. None of the single and dual task gait outcomes were found to have group × time interactions except for the cognitive DTCs of cadence (a borderline significant *p* = 0.018).

## Discussion

The goal of the current study was to assess the effects of VR-based cognitive and physical training on executive function and dual-task gait performance in older adults with MCI, as well as compare VR training with CPC training. In this study, we found significant improvements in executive function (SCWT), single-task gait performance and motor dual-task gait performance in both groups. However, only the VR group showed improvements in cognitive dual-task gait performance and the DTC of cadence after training. Moreover, the VR group showed more improvements than the CPC group in the TMT-B and cognitive DTC of cadence.

In our present study, the SCWT was used to assess selective attention or inhibition. Selective attention (inhibition) is the ability to control/inhibit impulsive responses and create responses by using attention and reasoning. This cognitive ability is one of our executive functions and contributes to anticipation, planning, and goal setting. Therefore, the stronger the inhibition ability is, the less interference will be produced by a new stimulus (Stroop, 1935). Falbo et al. (2016) state that physical and cognitive dual-task training effectively increased inhibitory performance in older adults. In the present dual-task intervention, subjects in both groups learned how to complete the task with multiple stimuli under the mutual interference of the cognitive and physical training. After repetitive practice, interference from new stimuli can be reduced, as reflected by improvements in inhibition shown by the SCWT in both groups.

The TMT-B assesses divided attention, which is the ability to attend to two different stimuli at the same time and respond to the multiple demands of the surroundings. The delta TMT assesses cognitive flexibility, which is an important aspect for dual tasking and prioritization during gait (Hobert et al., 2011, 2017). Divided attention and cognitive flexibility are also aspects of executive function, which allow us to process information from different sources and successfully carry out multiple tasks at a time. The effect of VR-based training on enhancing divided attention assessed *via* the TMT had been proved in individuals with Parkinson’s disease (Mirelman et al., 2011). In the present study, significant within- and between-group differences on the TMT-B were observed in older adults individuals with MCI who received VR training. We suggest that the improvements on the TMT-B might be attributed to the interaction of the VR programs. Our IADL-based VR programs effectively facilitated complex executive function, especially visual attention, as participants repetitively practiced these functional tasks during the 12-week intervention. For example, the kitchen chef game was specifically designed to train for planning and task switching, while participants prepared food as ordered with available kitchenware and ingredients. The convenience store clerk game trained for working memory, orientation and attention as the participants retrieved and calculated the prices of the checkout items. To optimize their performance, participants needed to increase both motor and cognitive capacity. We believe performing these functional tasks with internalized real-time feedback by VR may have a greater effect on various executive functions. Another explanation is that the enjoyment and attractiveness of the VR characteristics may have increased motivation and led to more extensive training effects on executive function in the VR group than the CPC group. Overall, the traditional combined physical and cognitive training program could not offer these critical features.

Both groups showed similar time effects on gait speed and stride length in the single-task and motor dual-task gait tests. These improvements imply the nonspecific exercise benefits of these two different interventions (Liberman et al., 2017). However, only the VR group showed an improvement in cognitive dual-task gait performance after the intervention. This gait improvement may have transferred from improvements in executive functions; in particular, we observed between-group differences in the TMT-B improvement, which represents visual attention and task switching. In fact, the role of executive function in walking when combined with a secondary task has been previously shown (Hausdorff et al., 2005; Schweiger et al., 2008). Furthermore, Parikh and Shah (2017) showed a relationship between the TMT-B and dual-task gait performance among older adults. Older adults with MCI have an increased fall risk compared to cognitively normal older adults (Liu-Ambrose et al., 2008). A gait velocity less than 1.0 m/s has also been associated with an increased fall risk among community-dwelling older adults (Abellan van Kan et al., 2009). Although both of our groups had improved cognitive and motor dual-task gait velocities after the intervention, the post-intervention gait velocities were still below 1.0 m/s in both cognitive and motor dual-task walking conditions. We suggest that sustained cognitive and physical training and fall prevention education are required in routine daily treatment programs.

The DTC quantifies dual-task interference, which is the relative change in performance associated with dual tasking. Individuals with cognitive deficits may be particularly susceptible to dual-task interference because there are fewer attentional resources available for the simultaneous performance of secondary tasks. We observed intragroup and intergroup improvements in the cognitive DTC of cadence after our VR training. The main cause may have been a specific effect of VR. VR not only provides a more complex environment but also creates many opportunities to train visual attention. From the concept of capacity-sharing theory, if two attention-demanding tasks are performed at the same time in a condition of limited attentional resources, performance of at least one of the tasks will deteriorate (Ruthruff et al., 2001; Yogev-Seligmann et al., 2008). We speculate that our VR training, including many IADL-based cognitive training components in augmented scenarios, might have improved cognitive capacity and reduced the attention needed to perform the cognitive task, thereby permitting greater attention to be shifted toward performing another concurrent task (e.g., walking). Therefore, the DTC of cadence were reduced from 11% to 6% after training.

Previous studies have stated that normal older adults can improve their cognitive dual-task gait performance after combined physical and cognitive training (Eggenberger et al., 2015; Falbo et al., 2016; Wang et al., 2018a). Contrary to our expectations, our CPC group showed no time effect on the cognitive dual-task walking performance. We suggest that for older adults with MCI, improvements in SCWT can transfer to improvements in motor dual-task performance but are not strong enough to transfer to improvements in cognitive dual-task performance (walking during serial subtraction). In fact, walking while doing serial subtraction is a highly demanding task that requires alternating momentary processing capacity and filtering out all signals that are irrelevant to counting itself. Hunter et al. (2018) proposed a framework for the secondary gait testing incorporating cognitive and motor tasks in older adults with MCI. According to this framework, the difficulty level of the cognitive dual task (serial subtractions) is higher than that of the motor dual task (carrying glass of water on tray) and more easily increases the cognitive costs in older adults with MCI (Hunter et al., 2018). Makizako et al. (2012) stated that applying multicomponent exercises had no significant effect on cognitive dual-task gait performance in older adults with MCI. Our findings are in agreement with these findings, although we added cognitive training to the physical exercise program. A higher intensity, duration and challenge might be required to transfer the cognitive dual-task skills to the gait performance in our CPC group.

To our knowledge, this study is the first to examine the effects of IADL-based VR training on dual-task gait performance in older adults with MCI. Limitations of this study include the lack of an actual control group such as placebo treatment or no intervention at all, which made the mechanisms underlying our results unclear. Second, the motor task we chose was too simple to create a challenging dual-task condition, so the training effects of the motor dual-task gait were similar to the single-task gait in both groups. Third, more gait parameters, such as gait variability and stride time, may be required in future studies, which might help us to clarify the mechanism of the improvements. Fourth, the dual-task program in the VR group was performed sequentially, and the dual-task program in the CPC group was performed simultaneously. Although most articles have stated that simultaneous and sequential dual-task training were both effective in improving cognition and led to similar effects (Strouwen et al., 2017; Bruderer-Hofstetter et al., 2018), whether a different combination of methods leads to different training intensities may require further investigation. Fifth, because of the large number of statistical tests and the small sample, the *p*-values cannot be interpreted in conventional terms as estimates of Type I and TYpe II error probability. They should be confirmed in a subsequent properly powered trial.

## Conclusion

The current results suggest that executive function and motor dual-task performance could benefit from both VR-based and traditional combined physical and cognitive training in older adults with MCI. However, VR-based physical and cognitive training showed more improvements in divided attention and the cognitive DTC than traditional combined physical and cognitive training. Our physical and cognitive program, derived from IADLs, may constitute a reference for the VR training effect in older adults with MCI.

## Data Availability

The raw data supporting the conclusions of this manuscript will be made available by the authors, without undue reservation, to any qualified researcher.

## Ethics Statement

The study protocol was approved by the Institutional Human Research Ethics Committee of National Yang-Ming University and has been registered at http://www.clinicaltrials.in.th/ (TCTR20180531001 on 1-October-2018). Written informed consent was obtained.

## Author Contributions

Y-YL conceived and designed the experiments. Y-YL, YC, and W-CH performed the experiments. Y-YL, I-HC, and W-CH analyzed the data. Y-YL, I-HC, and Y-JL wrote the article. All authors reviewed the manuscript.

## Conflict of Interest Statement

The authors declare that the research was conducted in the absence of any commercial or financial relationships that could be construed as a potential conflict of interest.

## Footnotes

^1^http://www.clinicaltrials.in.th/

## References

[B1] Arnett J. A. Labovitz S. S. (1995). Effect of physical layout in performance of the trail making test. Psychol. Assess. 7, 220–221. 10.1037/1040-3590.7.2.220

[B2] Abellan van KanG.RollandY.AndrieuS.BauerJ.BeauchetO.BonnefoyM.. (2009). Gait speed at usual pace as a predictor of adverse outcomes in community-dwelling older people an international academy on nutrition and aging (IANA) task force. J. Nutr. Health Aging 13, 881–889. 10.1007/s12603-009-0246-z19924348

[B3] Anderson-Hanley C. Barcelos N. M. Zimmerman E. A. Gillen R. W. Dunnam M. Cohen B. D.. (2018). The aerobic and cognitive exercise study (ACES) for community-dwelling older adults with or at-risk for mild cognitive impairment (MCI): neuropsychological, neurobiological and neuroimaging outcomes of a randomized clinical trial. Front. Aging Neurosci. 10:76. 10.3389/fnagi.2018.00076 29780318 PMC5945889

[B4] Bahureksa L. Najafi B. Saleh A. Sabbagh M. Coon D. Mohler M. J.. (2017). The impact of mild cognitive impairment on gait and balance: a systematic review and meta-analysis of studies using instrumented assessment. Gerontology 63, 67–83. 10.1159/000445831 27172932 PMC5107359

[B5] Barnes D. E. Santos-Modesitt W. Poelke G. Kramer A. F. Castro C. Middleton L. E.. (2013). The Mental Activity and eXercise (MAX) trial: a randomized controlled trial to enhance cognitive function in older adults. JAMA Intern. Med. 173, 797–804. 10.1001/jamainternmed.2013.189 23545598 PMC5921904

[B6] Bruderer-Hofstetter M. Rausch-Osthoff A. K. Meichtry A. Munzer T. Niedermann K. (2018). Effective multicomponent interventions in comparison to active control and no interventions on physical capacity, cognitive function and instrumental activities of daily living in elderly people with and without mild impaired cognition—A systematic review and network meta-analysis. Ageing Res. Rev. 45, 1–14. 10.1016/j.arr.2018.04.002 29679658

[B7] Chodzko-Zajko W. J. Proctor D. N. Fiatarone Singh M. A. Minson C. T. Nigg C. R. Salem G. J.. (2009). American College of Sports Medicine position stand. Exercise and physical activity for older adults. Med. Sci. Sports Exerc. 41, 1510–1530. 10.1249/MSS.0b013e3181a0c95c 19516148

[B8] Coyle H. Traynor V. Solowij N. (2015). Computerized and virtual reality cognitive training for individuals at high risk of cognitive decline: systematic review of the literature. Am. J. Geriatr. Psychiatry 23, 335–359. 10.1016/j.jagp.2014.04.009 24998488

[B9] Dadashi F. Mariani B. Rochat S. Büla C. J. Santos-Eggimann B. Aminian K. (2014). Gait and foot clearance parameters obtained using shoe-worn inertial sensors in a large-population sample of older adults. Sensors 14, 443–457. 10.3390/s140100443 24379049 PMC3926567

[B10] Damirchi A. Hosseini F. Babaei P. (2018). Mental training enhances cognitive function and BDNF more than either physical or combined training in elderly women with MCI: a small-scale study. Am. J. Alzheimers Dis. Other Demen. 33, 20–29. 10.1177/1533317517727068 28946752 PMC10852433

[B11] de Bruin E. D. Schmidt A. (2010). Walking behaviour of healthy elderly: attention should be paid. Behav. Brain Funct. 6:59. 10.1186/1744-9081-6-59 20939911 PMC2959004

[B12] Diamond A. (2013). Executive functions. Annu. Rev. Psychol. 64, 135–168. 10.1146/annurev-psych-113011-143750 23020641 PMC4084861

[B13] Doi T. Shimada H. Makizako H. Tsutsumimoto K. Uemura K. Anan Y.. (2014). Cognitive function and gait speed under normal and dual-task walking among older adults with mild cognitive impairment. BMC Neurol. 14:67. 10.1186/1471-2377-14-67 24694100 PMC3994221

[B14] Eggenberger P. Theill N. Holenstein S. Schumacher V. De Bruin E. D. (2015). Multicomponent physical exercise with simultaneous cognitive training to enhance dual-task walking of older adults: a secondary analysis of a 6-month randomized controlled trial with 1-year follow-up. Clin. Interv. Aging 10, 1711–1732. 10.2147/cia.s91997 26604719 PMC4631411

[B15] Falbo S. Condello G. Capranica L. Forte R. Pesce C. (2016). Effects of physical-cognitive dual task training on executive function and gait performance in older adults: a randomized controlled trial. Biomed Res. Int. 2016:5812092. 10.1155/2016/5812092 28053985 PMC5178854

[B17] Garber C. E. Blissmer B. Deschenes M. R. Franklin B. A. Lamonte M. J. Lee I. M.. (2011). American College of Sports Medicine position stand. Quantity and quality of exercise for developing and maintaining cardiorespiratory, musculoskeletal and neuromotor fitness in apparently healthy adults: guidance for prescribing exercise. Med. Sci. Sports Exerc. 43, 1334–1359. 10.1249/mss.0b013e318213fefb 21694556

[B18] García-Betances R. I. Jiménez-Mixco V. Arredondo M. T. Cabrera-Umpiérrez M. F. (2015). Using virtual reality for cognitive training of the elderly. Am. J. Alzheimers Dis. Other Demen. 30, 49–54. 10.1177/1533317514545866 25107931 PMC10852905

[B19] Hausdorff J. M. Yogev G. Springer S. Simon E. S. Giladi N. (2005). Walking is more like catching than tapping: gait in the elderly as a complex cognitive task. Exp. Brain Res. 164, 541–548. 10.1007/s00221-005-2280-3 15864565

[B20] Herman T. Mirelman A. Giladi N. Schweiger A. Hausdorff J. M. (2010). Executive control deficits as a prodrome to falls in healthy older adults: a prospective study linking thinking, walking, and falling. J. Gerontol. A Biol. Sci. Med. Sci. 65, 1086–1092. 10.1093/gerona/glq077 20484336 PMC2949331

[B21] Hobert M. A. Meyer S. I. Hasmann S. E. Metzger F. G. Suenkel U. Eschweiler G. W.. (2017). Gait is associated with cognitive flexibility: a dual-tasking study in healthy older people. Front. Aging Neurosci. 9:154. 10.3389/fnagi.2017.00154 28596731 PMC5442228

[B22] Hobert M. A. Niebler R. Meyer S. I. Brockmann K. Becker C. Huber H.. (2011). Poor trail making test performance is directly associated with altered dual task prioritization in the elderly–baseline results from the TREND study. PLoS One 6:e27831. 10.1371/journal.pone.0027831 22114705 PMC3218043

[B23] Hunter S. W. Divine A. Frengopoulos C. Montero-Odasso M. (2018). A framework for secondary cognitive and motor tasks in dual-task gait testing in people with mild cognitive impairment. BMC Geriatr. 18:202. 10.1186/s12877-018-0894-0 30176796 PMC6122701

[B24] Johns E. K. Phillips N. A. Belleville S. Goupil D. Babins L. Kelner N.. (2012). The profile of executive functioning in amnestic mild cognitive impairment: disproportionate deficits in inhibitory control. J. Int. Neuropsychol. Soc. 18, 541–555. 10.1017/s1355617712000069 22370245

[B25] Johnson J. K. Lui L. Y. Yaffe K. (2007). Executive function, more than global cognition, predicts functional decline and mortality in elderly women. J. Gerontol. A Biol. Sci. Med. Sci. 62, 1134–1141. 10.1093/gerona/62.10.1134 17921427 PMC2049089

[B26] Kirova A. M. Bays R. B. Lagalwar S. (2015). Working memory and executive function decline across normal aging, mild cognitive impairment and Alzheimer’s disease. Biomed Res. Int. 2015:748212. 10.1155/2015/748212 26550575 PMC4624908

[B27] Liberman K. Forti L. N. Beyer I. Bautmans I. (2017). The effects of exercise on muscle strength, body composition, physical functioning and the inflammatory profile of older adults: a systematic review. Curr. Opin. Clin. Nutr. Metab. Care 20, 30–53. 10.1097/mco.0000000000000335 27755209

[B28] Liu-Ambrose T. Y. Ashe M. C. Graf P. Beattie B. L. Khan K. M. (2008). Increased risk of falling in older community-dwelling women with mild cognitive impairment. Phys. Ther. 88, 1482–1491. 10.2522/ptj.20080117 18820094 PMC3514550

[B29] Makizako H. Doi T. Shimada H. Yoshida D. Tsutsumimoto K. Uemura K.. (2012). Does a multicomponent exercise program improve dual-task performance in amnestic mild cognitive impairment? A randomized controlled trial. Aging Clin. Exp. Res. 24, 640–646. 10.3275/8760 23211228

[B30] Mariani B. Hoskovec C. Rochat S. Büla C. Penders J. Aminian K. (2010). 3D gait assessment in young and elderly subjects using foot-worn inertial sensors. J. Biomech. 43, 2999–3006. 10.1016/j.jbiomech.2010.07.003 20656291

[B31] McDowd J. M. (1986). The effects of age and extended practice on divided attention performance. J. Gerontol. 41, 764–769. 10.1093/geronj/41.6.764 3772053

[B32] Mirelman A. Maidan I. Herman T. Deutsch J. E. Giladi N. Hausdorff J. M. (2011). Virtual reality for gait training: can it induce motor learning to enhance complex walking and reduce fall risk in patients with Parkinson’s disease? J. Gerontol. A Biol. Sci. Med. Sci. 66, 234–240. 10.1093/gerona/glq201 21106702

[B33] Montero-Odasso M. M. Sarquis-Adamson Y. Speechley M. Borrie M. J. Hachinski V. C. Wells J.. (2017). Association of dual-task gait with incident dementia in mild cognitive impairment: results from the gait and brain study. JAMA Neurol. 74, 857–865. 10.1001/jamaneurol.2017.0643 28505243 PMC5710533

[B34] Mrakic-Sposta S. Di Santo S. G. Franchini F. Arlati S. Zangiacomi A. Greci L.. (2018). Effects of combined physical and cognitive virtual reality-based training on cognitive impairment and oxidative stress in MCI patients: a pilot study. Front. Aging Neurosci. 10:282. 10.3389/fnagi.2018.00282 30327596 PMC6174250

[B35] Muir S. W. Speechley M. Wells J. Borrie M. Gopaul K. Montero-Odasso M. (2012). Gait assessment in mild cognitive impairment and Alzheimer’s disease: the effect of dual-task challenges across the cognitive spectrum. Gait Posture 35, 96–100. 10.1016/j.gaitpost.2011.08.014 21940172

[B36] Parikh H. S. K. Shah C. (2017). Relationship between executive function and dual task physical performance among older adults-a cross sectional study. Int. J. Phys. Med. Rehabil. 5:421 10.4172/2329-9096.1000421

[B37] Plummer P. Zukowski L. A. Giuliani C. Hall A. M. Zurakowski D. (2015). Effects of physical exercise interventions on gait-related dual-task interference in older adults: a systematic review and meta-analysis. Gerontology 62, 94–117. 10.1159/000371577 25721432

[B38] Rosso A. L. Metti A. L. Faulkner K. Redfern M. Yaffe K. Launer L.. (2019). Complex walking tasks and risk for cognitive decline in high functioning older adults. J. Alzheimers Dis. [Epub ahead of print]. 10.3233/jad-181140 30814353 PMC6703970

[B39] Ruthruff E. Pashler H. E. Klaassen A. (2001). Processing bottlenecks in dual-task performance: structural limitation or strategic postponement? Psychon. Bull. Rev. 8, 73–80. 10.3758/bf03196141 11340869

[B40] Scarpina F. Tagini S. (2017). The stroop color and word test. Front. Psychol. 8:557. 10.3389/fpsyg.2017.00557 28446889 PMC5388755

[B41] Schweiger A. Yogev-Seligmann G. Hausdorff J. M. Giladi N. Herman T. (2008). Dual-task decrements in gait: contributing factors among healthy older adults. J. Gerontol. A Biol. Sci. Med. Sci. 63, 1335–1343. 10.1093/gerona/63.12.1335 19126846 PMC3181497

[B42] Simon S. S. Yokomizo J. E. Bottino C. M. (2012). Cognitive intervention in amnestic Mild Cognitive Impairment: a systematic review. Neurosci. Biobehav. Rev. 36, 1163–1178. 10.1016/j.neubiorev.2012.01.007 22322184

[B43] Springer S. Giladi N. Peretz C. Yogev G. Simon E. S. Hausdorff J. M. (2006). Dual-tasking effects on gait variability: the role of aging, falls and executive function. Mov. Disord. 21, 950–957. 10.1002/mds.20848 16541455

[B44] Stroop J. R. (1935). Studies of interference in serial verbal reactions. J. Exp. Psychol. 18, 643–662. 10.1037/h0054651

[B45] Strouwen C. Molenaar E. Munks L. Keus S. H. J. Zijlmans J. C. M. Vandenberghe W.. (2017). Training dual tasks together or apart in Parkinson’s disease: results from the DUALITY trial. Mov. Disord. 32, 1201–1210. 10.1002/mds.27014 28440888

[B46] Suzuki T. Shimada H. Makizako H. Doi T. Yoshida D. Ito K.. (2013). A randomized controlled trial of multicomponent exercise in older adults with mild cognitive impairment. PLoS One 8:e61483. 10.1371/journal.pone.0061483 23585901 PMC3621765

[B47] Suzuki T. Shimada H. Makizako H. Doi T. Yoshida D. Tsutsumimoto K.. (2012). Effects of multicomponent exercise on cognitive function in older adults with amnestic mild cognitive impairment: a randomized controlled trial. BMC Neurol. 12:128. 10.1186/1471-2377-12-128 23113898 PMC3534485

[B48] Tay L. Lim W. S. Chan M. Ali N. Chong M. S. (2016). A combined cognitive stimulation and physical exercise programme (MINDVital) in early dementia: differential effects on single- and dual-task gait performance. Gerontology 62, 604–610. 10.1159/000444084 26913768

[B49] Tsai C. F. Lee W. J. Wang S. J. Shia B. C. Nasreddine Z. Fuh J. L. (2012). Psychometrics of the Montreal Cognitive Assessment (MoCA) and its subscales: validation of the Taiwanese version of the MoCA and an item response theory analysis. Int. Psychogeriatr. 24, 651–658. 10.1017/s1041610211002298 22152127

[B50] Wang R.-Y. Wang Y.-L. Cheng F.-Y. Chao Y.-H. Chen C.-L. Yang Y.-R. (2018a). Effects of a multicomponent exercise on dual-task performance and executive function among older adults. Int. J. Gerontol. 12, 133–138. 10.1016/j.ijge.2018.01.004

[B51] Wang R.-Y. Zhou J.-H. Huang Y.-C. Yang Y.-R. (2018b). Reliability of the chinese version of the trail making test and stroop color and word test among older adults. Int. J. Gerontol. 12, 336–339. 10.1016/j.ijge.2018.06.003

[B52] Woollacott M. Shumway-Cook A. (2002). Attention and the control of posture and gait: a review of an emerging area of research. Gait Posture 16, 1–14. 10.1016/s0966-6362(01)00156-4 12127181

[B53] Yogev-Seligmann G. Hausdorff J. M. Giladi N. (2008). The role of executive function and attention in gait. Mov. Disord. 23, 329–342; quiz 472. 10.1002/mds.21720 18058946 PMC2535903

